# Development and external validation of an explainable machine learning model for in-hospital mortality risk stratification in intensive care unit patients with heart failure

**DOI:** 10.3389/fcvm.2026.1942393

**Published:** 2026-09-04

**Authors:** Liusheng Xu, Wei Cao, Zefan Huang, Kefeng Lu, Yichen Fang, Jinhui Zhang, Ping Zhang, Tongbao Feng

**Affiliations:** 1 Department of Clinical Laboratory, The Second People’s Hospital of Changzhou, the Third Affiliated Hospital of Nanjing Medical University, Changzhou, Jiangsu, China; 2 Department of Urology, The Second People’s Hospital of Changzhou, the Third Affiliated Hospital of Nanjing Medical University, Changzhou, Jiangsu, China; 3 Institute of Reproductive Medicine, Medical School, Nantong University, Nantong, Jiangsu, China; 4 The First Clinical College, Xuzhou Medical University, Xuzhou, China; 5 Department of Critical Care Medicine, The Affiliated Hospital of Jiangsu University, Zhenjiang, Jiangsu, China

**Keywords:** explainable machine learning, external validation, heart failure, in-hospital mortality, intensive care unit

## Abstract

**Background:**

Early risk stratification for in-hospital mortality remains challenging in intensive care unit (ICU) patients with heart failure (HF) because of substantial clinical heterogeneity and complex pathophysiological interactions. Explainable machine learning (ML) may offer a practical and transparent approach to prognostic assessment in this high-risk population.

**Methods:**

This retrospective dual-cohort study included 18,526 ICU patients with HF from the MIMIC-IV database (2008–2022) as the development cohort and 314 consecutive ICU patients with HF from an independent hospital-based cohort from August 1, 2023, to May 31, 2026, as the external validation cohort. Candidate predictors available within 24 h of ICU admission were screened using the Boruta algorithm and least absolute shrinkage and selection operator regression. Five ML algorithms were developed in the development cohort and compared using 5-fold nested cross-validation for internal validation. Model interpretability was assessed using Shapley Additive Explanations (SHAP).

**Results:**

Nine routinely available predictors were retained for final model development. Among the candidate algorithms, Extreme Gradient Boosting (XGBoost) showed the most balanced overall performance and was selected as the final model. In internal validation using nested cross-validation, the XGBoost model achieved the highest validation area under the receiver operating characteristic curve (AUC), at 0.704 (95% CI 0.682–0.726). In the external validation cohort, the final model achieved an AUC of 0.877 (95% CI 0.820–0.933) and a Brier score of 0.079 (95% CI 0.061–0.099). However, this apparently stronger discrimination should be interpreted cautiously in light of cohort differences and the limited number of external events. SHAP analysis identified blood urea nitrogen, age, and white blood cell count as the most influential predictors.

**Conclusion:**

An explainable XGBoost-based model using nine routinely available early clinical variables showed promising performance for predicting in-hospital all-cause mortality in ICU patients with HF. This interpretable tool may support early risk stratification and individualized clinical management in critically ill patients with HF. Further large multicenter external validation is warranted.

## 1 Introduction

Heart failure (HF) is a cardiovascular syndrome characterized by substantial clinical heterogeneity and poor prognosis, affecting more than 64 million people worldwide and remaining associated with a high burden of hospitalization and mortality (1, 2). Among patients with HF admitted to the intensive care unit (ICU), the pathophysiological state is often more complex because of frequent hemodynamic disturbances, organ dysfunction, and metabolic imbalance, making short-term clinical assessment and risk stratification more challenging (3–8). Accurate early risk stratification based on clinical information available at admission has therefore become an important priority for optimizing monitoring strategies and improving in-hospital outcomes.

In critically ill patients with HF, the risk of in-hospital mortality is shaped by multiple interacting clinical and pathophysiological processes, including hemodynamic instability, cardiorenal dysfunction, inflammatory activation, coagulation abnormalities, and metabolic stress (4, 5, 9–11). This complexity makes prognostic assessment particularly challenging and may limit the ability of conventional approaches to fully capture the nonlinear relationships and higher-order interactions that underlie adverse outcomes (3, 12). Consequently, increasing attention has been directed toward prognostic modeling in patients with HF, particularly in the critical care setting. Compared with traditional statistical methods, machine learning (ML) approaches can simultaneously process large numbers of clinical variables and model potential nonlinear relationships and complex interactions, thereby offering potential advantages for risk stratification in heterogeneous ICU populations. Previous studies based on critical care databases or multicenter cohorts have applied algorithms such as random forest (RF), Extreme Gradient Boosting (XGBoost), support vector machines, and neural networks to predict in-hospital mortality, short-term readmission, and other adverse outcomes in patients with HF. These studies have generally reported encouraging discriminatory performance (6–8, 13–15). Nevertheless, several important limitations remain in the current literature. Some published models rely on a large number of predictors or complex scoring procedures, which may limit their practicality in routine clinical practice (15–17). In addition, some studies have been validated only internally within a single dataset and have lacked independent external validation, thereby weakening confidence in their generalizability and robustness. Furthermore, evidence regarding the key predictors of in-hospital mortality and their contribution patterns in ICU patients with HF remains limited, particularly for models intended to support early bedside risk assessment (18–21). Previously, we reported that the cholesterol, high-density lipoprotein, and glucose (CHG) index, a composite biomarker derived from routine laboratory tests, was independently associated with in-hospital all-cause mortality in patients with HF. While that study focused on the prognostic value of a single biomarker, critically ill patients with HF often present with multiple interacting abnormalities that may not be adequately captured by any single indicator alone (22).

In this context, explainable machine learning may offer value beyond prediction alone. Although machine learning methods are well suited to modeling complex clinical data, their clinical adoption may be limited if model outputs cannot be interpreted in a transparent and clinically meaningful manner. Explainable machine learning methods, particularly Shapley Additive Explanations (SHAP), provide a useful framework for quantifying the contribution of individual variables to model predictions and for illustrating how specific clinical features increase or decrease predicted risk at both the population and individual levels (23–25). Such interpretability may enhance model transparency, improve clinicians’ understanding of risk drivers, and support more credible and clinically applicable prediction in this high-risk population.

Accordingly, this study aimed to develop and externally validate an explainable machine learning model for predicting in-hospital all-cause mortality in ICU patients with HF. Using the Medical Information Mart for Intensive Care IV (MIMIC-IV, version 3.1) database as the development cohort and an independent hospital-based cohort for external validation, we compared the predictive performance of multiple ML algorithms, identified the optimal model, and applied SHAP to characterize the relative importance of key predictors and their contribution patterns. Our goal was to provide a practical, interpretable tool to support early risk stratification and individualized clinical management in critically ill patients with HF.

## 2 Methods

### 2.1 Study design and data sources

This retrospective observational study used a dual-cohort design to develop and externally validate a machine learning-based model for predicting in-hospital all-cause mortality in ICU patients with HF. The study was conducted in accordance with the Declaration of Helsinki and reported in accordance with the Transparent Reporting of a multivariable prediction model for Individual Prognosis Or Diagnosis plus Artificial Intelligence (TRIPOD + AI) statement, the updated reporting guidance for clinical prediction models that use regression or machine learning methods (26). A completed TRIPOD + AI checklist is provided in Supplementary File S1.

Patients in the development cohort were identified from the MIMIC-IV database (version 3.1), a publicly available de-identified critical care database containing fully de-identified electronic health records from Beth Israel Deaconess Medical Center (BIDMC). This resource includes ICU admissions recorded between 2008 and 2022 and provides comprehensive information on patient demographics, bedside vital signs, laboratory measurements, comorbidities, treatment-related variables, and clinical outcomes. The BIDMC Institutional Review Board approved the use of the de-identified data for research and waived the requirement for informed consent (27). Access to MIMIC-IV is restricted to researchers who complete the required credentialing and authorization procedures through PhysioNet and sign a Data Use Agreement. The database used in this study was accessed by Jinhui Zhang on April 7, 2025. The available Collaborative Institutional Training Initiative completion record for this authorized user was record ID 65008026 (completed September 8, 2024; expires September 8, 2028).

The external validation cohort was identified from the Electronic Medical Record System (EMRS) and Laboratory Information System (LIS) of the Third Affiliated Hospital of Nanjing Medical University and included consecutive adult patients with HF admitted to the ICU from August 1, 2023, to May 31, 2026. The protocol for the hospital-based cohort was approved by the Clinical Research Ethics Committee of the Third Affiliated Hospital of Nanjing Medical University [approval number: (2023) KY204-01]. Given the retrospective nature of the study and the use of de-identified clinical data, the requirement for informed consent was waived.

### 2.2 Study participants and outcome definition

In the MIMIC-IV database, adult patients admitted to the ICU were first screened, and cases of HF were subsequently identified based on International Classification of Diseases, Ninth and Tenth Revisions (ICD-9 and ICD-10) codes and hospitalization diagnosis records (28). For patients with multiple ICU admissions, only data from the first ICU admission were retained to reduce bias related to repeated measurements.

**Figure 1:**
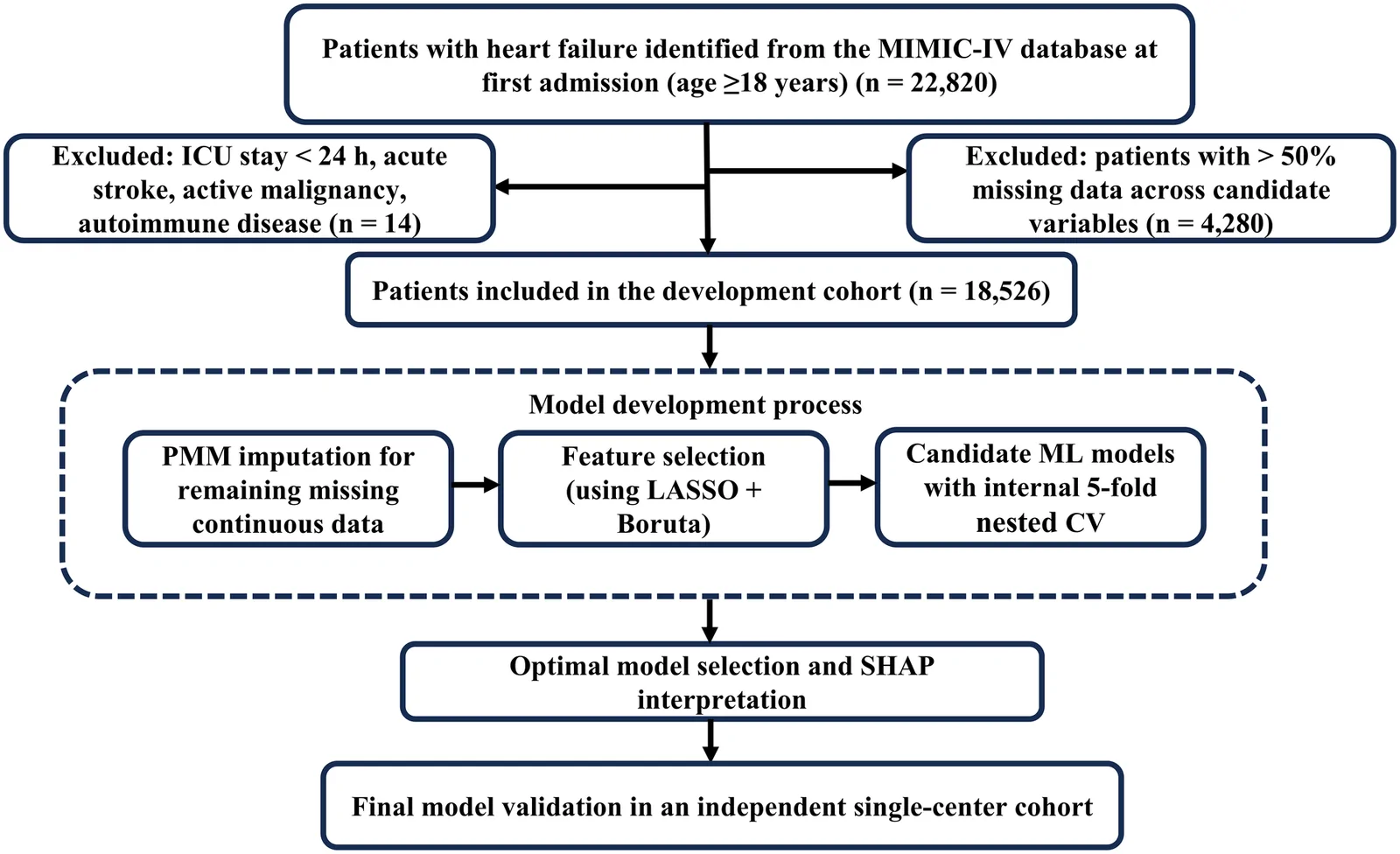
Flowchart of patient selection and model development.

The inclusion criteria were as follows: (1) age ≥18 years; (2) first ICU admission; (3) a diagnosis of HF; and (4) definite in-hospital outcome information. Cohort assembly was performed sequentially according to the prespecified eligibility criteria. The exclusion criteria were as follows: (1) ICU stay <24 h; and (2) coexisting acute stroke, active malignancy, or autoimmune disease. These conditions were excluded because they may independently influence short-term in-hospital mortality through non-HF-related mechanisms and may also substantially affect key early clinical indicators, such as inflammatory, coagulation, renal, and hemodynamic parameters, thereby increasing clinical heterogeneity and potentially confounding the assessment of HF-related risk (29–31). Because these disease-related exclusions were applied after prior cohort restrictions had already been used to define the eligible HF ICU cohort, the corresponding excluded count reflects sequential cohort refinement rather than the prevalence of these conditions in the full MIMIC-IV source population. The external validation cohort used the same inclusion and exclusion criteria as the development cohort and consecutively enrolled adult patients admitted to the ICU during the study period who met the diagnostic criteria for HF. The patient selection process is shown in Figure 1.

The primary outcome of this study was in-hospital all-cause mortality, defined as death from any cause occurring during the index hospitalization included in the study. Outcome data for the development cohort were obtained from the in-hospital outcome records in the MIMIC-IV database, whereas outcome data for the external validation cohort were obtained from the EMRS of the Third Affiliated Hospital of Nanjing Medical University.

### 2.3 Candidate predictors

Candidate predictors were prespecified based on clinical availability, evidence from previous literature, and potential clinical relevance (19, 32, 33). These variables included the following categories: (1) Demographics: age and sex; (2) Comorbidities: diabetes, hypertension, previous myocardial infarction, chronic pulmonary disease, and renal disease; (3) Vital signs: heart rate, systolic blood pressure (SBP), diastolic blood pressure (DBP), and oxygen saturation (SpO_2_); and (4) Laboratory tests: blood glucose, serum creatinine (SCr), blood urea nitrogen (BUN), red blood cell count (RBC), hemoglobin (Hb), white blood cell count (WBC), platelet count (PLT), prothrombin time (PT), potassium, and sodium.

For continuous variables with repeated measurements, the first value recorded within 24 h after ICU admission was extracted for model development. For baseline comorbidities, variables in the development cohort were identified from diagnosis information recorded in the MIMIC-IV database, whereas those in the external validation cohort were obtained from the hospital EMRS according to documented clinical diagnoses. For the remaining variables, the external validation cohort used the same variable definitions, data extraction window, and unit conversion rules as the development cohort to improve cross-cohort comparability. The relevant unit conversion rules are provided in Supplementary Table S1.

### 2.4 Data preprocessing and feature selection

Before model development, candidate variables were systematically examined for missingness, distributional characteristics, and outliers. Before imputation, records with substantial individual-level missingness across the broader set of initially extracted variables were excluded, as they contained insufficient information for reliable data recovery and subsequent model development. The >50% missingness criterion referred to patient-level missingness across this initial variable pool before candidate-variable reduction, rather than to per-variable missingness in the final candidate predictor set. The proportions of missing data for candidate continuous variables in the development cohort are summarized in Supplementary Table S2. The highest missingness among continuous candidate variables was 6.27%. No missing values were identified in the categorical variables included in the analysis. Missing continuous variables in the development cohort were imputed using predictive mean matching (PMM) with 5 imputations (m = 5). For subsequent feature selection and model development, one completed dataset generated from the multiple-imputation procedure was used (34). As no missing values were identified among variables in the external validation cohort, missing-data imputation was not performed.

To reduce variable redundancy and improve model robustness, feature selection was performed using a combination of the Boruta algorithm and least absolute shrinkage and selection operator (LASSO) regression (24, 35). First, the Boruta algorithm identified outcome-related variables based on random forest-derived importance measures. Subsequently, LASSO regression was applied to further reduce dimensionality, and the optimal penalty parameter was determined by 10-fold cross-validation. To balance variable importance and model parsimony while minimizing the impact of redundant predictors on model stability, only variables retained by both Boruta and LASSO were included in subsequent model construction. All preprocessing and feature-selection procedures were performed in the development cohort before model training and development.

### 2.5 Model development and external validation

In the development cohort, logistic regression (LR), Light Gradient Boosting Machine (LightGBM), Gaussian Naive Bayes (GNB), XGBoost, and RF models were developed using the variables retained after feature selection.

A 5-fold nested cross-validation (CV) framework combined with grid search was used for model training, hyperparameter optimization, and internal validation (7, 19). A fixed random seed of 42 was used for the nested CV splitting procedure to improve reproducibility. In each outer-loop iteration, one fold was held out as the validation set and the remaining folds were used for model training. Within each outer-loop training set, an inner 5-fold CV combined with grid search was performed for hyperparameter tuning. The prespecified hyperparameter search space for each candidate machine learning algorithm is summarized in Supplementary Table S3. Performance comparison among candidate algorithms was based on the pooled outer-loop validation results. Based on the nested CV results, the algorithm with the best overall performance was selected as the final modeling framework. Subsequently, after the optimal algorithm had been selected, the hyperparameter settings within this framework were further refined based on the grid search results and performance in nested CV to improve model stability and reduce the risk of overfitting.

Because the outcome showed a certain degree of class imbalance but was not severely imbalanced, resampling methods such as the Synthetic Minority Oversampling Technique (SMOTE) were not used, in order to preserve the original distribution of clinical outcomes and avoid additional noise potentially introduced by oversampling (36). During model development, the cohort was not further divided into fixed training and test sets; instead, all 18,526 patients in the development cohort were included in the nested CV procedure. Compared with a single random split, nested CV made more complete use of the information available in the development cohort and provided relatively robust internal validation during model comparison and hyperparameter optimization (37).

Finally, the model was retrained on the entire development cohort using the selected algorithmic framework, retained feature set, and the final hyperparameter settings, and was then evaluated in the independent external validation cohort from the Third Affiliated Hospital of Nanjing Medical University. The external validation cohort was not involved in feature selection, model training, or hyperparameter refinement.

### 2.6 Statistical analysis and model interpretation

For descriptive analyses, the distribution of continuous variables was evaluated using the Shapiro–Wilk test. Variables with a normal distribution are presented as the mean ± standard deviation, and between-group comparisons were performed using the Student's t-test or analysis of variance (ANOVA). Variables with a non-normal distribution are described as the median with interquartile range (IQR) and were compared using the Mann–Whitney *U* test. Categorical variables are summarized as counts and percentages, and comparisons between groups were conducted using the *χ*^2^ test or Fisher's exact test.

Within the development cohort, model performance was summarized using the pooled outer-loop validation results from the 5-fold nested CV. Model comparison metrics primarily included the area under the receiver operating characteristic curve (AUC) and its 95% confidence interval (CI), accuracy, sensitivity, specificity, positive predictive value (PPV), negative predictive value (NPV), and F1 score. The primary internal comparison among candidate algorithms was based on the outer-loop validation results. For the finalized model after post-selection hyperparameter refinement, the reported 95% CI for the AUC was obtained from fold-level confidence intervals estimated using DeLong's method and then summarized across the 5-fold nested cross-validation results. Calibration was assessed graphically using decile-binned calibration plots and quantitatively using the Brier score, calibration slope, and calibration-in-the-large (CITL). Clinical utility was evaluated by decision curve analysis (DCA) across a range of threshold probabilities. In addition, to further contextualize the performance of the proposed model, two established ICU severity scores, the Sequential Organ Failure Assessment (SOFA) score and the Acute Physiology Score III (APS III), were additionally evaluated in the development cohort using variables obtained within 24 h after ICU admission, and their discriminatory ability and clinical net benefit were compared with those of the final model.

During independent external validation, model performance was assessed in terms of discrimination, calibration, overall prediction error, and clinical utility, using the AUC, calibration plots, the Brier score, and DCA, respectively. The optimal probability cutoff was determined during model development according to the principle of maximizing the Youden index and was then fixed and applied to the external validation cohort for binary classification purposes. However, clinical utility was additionally assessed across a range of threshold probabilities using DCA rather than being inferred from this single cutoff alone. On this basis, predicted probabilities were converted into binary classification results to calculate accuracy, sensitivity, specificity, PPV, NPV, and F1 score.

To improve model interpretability, SHAP analysis was performed in the development cohort to quantify the contribution of each variable to model predictions, and the direction and relative importance of key variables were illustrated using global feature importance plots, beeswarm plots, and individual explanation plots. Restricted cubic spline (RCS) analyses were additionally performed in the development cohort for the continuous variables retained in the final model to explore their potential nonlinear associations with in-hospital mortality. The corresponding dose-response curves are presented as odds ratios (ORs) with 95% CIs. All data processing, statistical analyses, and model development were conducted in R and Python. The main software packages used for model development were R 4.2.2 (including the mice 3.19.0, glmnet 4.1.8, and Boruta 9.0.0 packages) and Python 3.9 (including scikit-learn 1.0.2, xgboost 1.6.1, lightgbm 3.3.2, and shap 0.43.0). In addition, a web-based calculator implementing the final model is available online, and the final trained model is maintained in a serialized.pkl format for research purposes.

## 3 Results

### 3.1 Baseline characteristics

A total of 18,840 patients were included in this study, including 18,526 in the MIMIC-IV development cohort, of whom 2,986 died during hospitalization (16.12%), and 314 in the external validation cohort, of whom 36 died during hospitalization (11.46%).

Table 1 presents the baseline characteristics of the development and external validation cohorts. No significant differences were observed between the two cohorts in terms of age or sex distribution. The median age was 73.95 years (IQR, 64.18–82.68) in the development cohort and 76.00 years (IQR, 67.00–82.00) in the external validation cohort (*p* = 0.637); the proportion of women was 43.84% in the development cohort and 40.13% in the external validation cohort (*p* = 0.188). Compared with the development cohort, the external validation cohort had a higher prevalence of hypertension and lower prevalences of previous myocardial infarction and chronic pulmonary disease (all *p* < 0.001). For vital signs, heart rate, SBP, and DBP were higher in the external validation cohort. For laboratory tests, RBC, Hb, glucose, and sodium were higher, whereas WBC, PLT, SCr, PT, and potassium were lower (all *p* < 0.05). These findings indicate differences in case mix and clinical characteristics between the two cohorts.

**Table 1:** Baseline characteristics of the MIMIC-IV development cohort and the external validation cohort.

Variables	Development cohort (*n* = 18,526)	External validation cohort (*n* = 314)	*P* value
**Demographics**			
Age, years	73.95 (64.18, 82.68)	76.00 (67.00, 82.00)	0.637
Sex, n (%)	0.188		
Female	8122 (43.84)	126 (40.13)	
Male	10404 (56.16)	188 (59.87)	
**Comorbidities**			
Hypertension, n (%)	4093 (22.09)	235 (74.84)	<0.001
Diabetes, n (%)	8242 (44.49)	146 (46.50)	0.478
Prior myocardial infarction, n (%)	6125 (33.06)	41 (13.06)	<0.001
Renal disease, n (%)	8065 (43.53)	135 (42.99)	0.848
Chronic pulmonary disease, n (%)	6953 (37.53)	18 (5.73)	<0.001
**Vital signs**			
Heart rate, bpm	86.00 (75.00, 101.00)	93.00 (81.00, 107.00)	<0.001
SBP, mmHg	119.00 (104.00, 137.00)	138.00 (120.00, 158.00)	<0.001
DBP, mmHg	65.00 (54.00, 77.00)	79.00 (67.00, 92.00)	<0.001
SpO_2_, %	98.00 (95.00, 100.00)	97.00 (95.00, 99.00)	0.006
**Laboratory measurements**			
RBC, *10^12^/L	3.47 (2.94, 4.05)	3.88 (3.25, 4.51)	<0.001
Hb, g/dL	10.50 (8.90, 12.20)	11.60 (9.60, 13.40)	<0.001
WBC, *10^9^/L	10.50 (7.50, 14.80)	8.51 (6.09, 11.60)	<0.001
PLT, *10^9^/L	193.00 (141.00, 259.00)	181.00 (136.00, 235.00)	0.006
SCr, mg/dL	1.30 (0.90, 2.20)	1.19 (0.86, 1.90)	0.003
BUN, mg/dL	28.00 (18.00, 46.00)	27.17 (18.77, 43.42)	0.827
Glucose, mg/dL	130.00 (104.00, 174.00)	138.57 (107.76, 198.22)	0.010
PT, s	14.90 (12.90, 18.80)	12.90 (11.90, 14.40)	<0.001
Potassium, mmol/L	4.20 (3.80, 4.70)	4.01 (3.62, 4.45)	<0.001
Sodium, mmol/L	138.00 (135.00, 141.00)	140.00 (137.30, 142.80)	<0.001
**Outcome**			
In-hospital mortality, n (%)	2986 (16.12)	36 (11.46)	

SBP, systolic blood pressure; DBP, diastolic blood pressure; SpO_2_, oxygen saturation; RBC, red blood cell count; Hb, hemoglobin; WBC, white blood cell count; PLT, platelet count; SCr, serum creatinine; BUN, blood urea nitrogen; PT, prothrombin time.

Table 2 compares the baseline characteristics of survivors and nonsurvivors in the development cohort. Compared with survivors, nonsurvivors were older and had higher prevalences of previous myocardial infarction and renal disease, but a lower prevalence of hypertension (all *p* < 0.001). For vital signs, nonsurvivors had a higher heart rate but lower SBP, DBP, and SpO_2_ (all *p* < 0.001). For laboratory tests, nonsurvivors had lower RBC, Hb, and PLT levels but higher WBC, SCr, BUN, PT, glucose, and potassium levels (all *p* < 0.001).

**Table 2:** Baseline characteristics of survivors and nonsurvivors in the MIMIC-IV development cohort.

Variables	Overall (*n* = 18,526)	Survivors (*n* = 15,540)	Nonsurvivors (*n* = 2,986)	*P* value
**Demographics**				
Age, years	73.95 (64.18, 82.68)	73.21 (63.41, 82.09)	77.58 (68.66, 85.15)	<0.001
Sex, n (%)				0.632
Female	8122 (43.84)	6801 (43.76)	1321 (44.24)	
Male	10404 (56.16)	8739 (56.24)	1665 (55.76)	
**Comorbidities**				
Hypertension, n (%)	4093 (22.09)	3596 (23.14)	497 (16.64)	<0.001
Diabetes, n (%)	8242 (44.49)	6945 (44.69)	1297 (43.44)	0.206
Prior myocardial infarction, n (%)	6125 (33.06)	5028 (32.36)	1097 (36.74)	<0.001
Renal disease, n (%)	8065 (43.53)	6547 (42.13)	1518 (50.84)	<0.001
Chronic pulmonary disease, n (%)	6953 (37.53)	5851 (37.65)	1102 (36.91)	0.441
**Vital signs**				
Heart rate, bpm	86.00 (75.00, 101.00)	85.00 (74.00, 99.00)	90.00 (76.00, 107.00)	<0.001
SBP, mmHg	119.00 (104.00, 137.00)	120.00 (105.00, 138.00)	114.00 (99.00, 131.00)	<0.001
DBP, mmHg	65.00 (54.00, 77.00)	65.00 (54.00, 78.00)	63.00 (52.00, 76.00)	<0.001
SpO_2_, %	98.00 (95.00, 100.00)	98.00 (95.00, 100.00)	97.00 (94.00, 100.00)	<0.001
**Laboratory measurements**				
RBC, *10^12^/L	3.47 (2.94, 4.05)	3.48 (2.96, 4.06)	3.39 (2.88, 3.97)	<0.001
Hb, g/dL	10.50 (8.90, 12.20)	10.70 (9.00, 12.30)	10.00 (8.50, 11.60)	<0.001
WBC, *10^9^/L	10.50 (7.50, 14.80)	10.30 (7.40, 14.40)	12.00 (8.40, 17.10)	<0.001
PLT, *10^9^/L	193.00 (141.00, 259.00)	194.00 (143.00, 258.00)	188.00 (128.00, 263.00)	<0.001
SCr, mg/dL	1.30 (0.90, 2.20)	1.30 (0.90, 2.00)	1.70 (1.10, 2.80)	<0.001
BUN, mg/dL	28.00 (18.00, 46.00)	27.00 (18.00, 44.00)	38.00 (24.00, 60.00)	<0.001
Glucose, mg/dL	130.00 (104.00, 174.00)	128.00 (103.00, 171.00)	139.00 (108.00, 189.00)	<0.001
PT, s	14.90 (12.90, 18.80)	14.70 (12.80, 18.20)	16.40 (13.60, 23.10)	<0.001
Potassium, mmol/L	4.20 (3.80, 4.70)	4.20 (3.80, 4.70)	4.30 (3.90, 4.90)	<0.001
Sodium, mmol/L	138.00 (135.00, 141.00)	138.00 (135.00, 141.00)	138.00 (134.00, 141.00)	0.007

SBP, systolic blood pressure; DBP, diastolic blood pressure; SpO_2_, oxygen saturation; RBC, red blood cell count; Hb, hemoglobin; WBC, white blood cell count; PLT, platelet count; SCr, serum creatinine; BUN, blood urea nitrogen; PT, prothrombin time.

Supplementary Table S4 further compares the nine selected predictors between survivors and nonsurvivors in the external validation cohort. Compared with survivors, nonsurvivors were older and had higher WBC, BUN, PT, glucose, and potassium levels, but lower Hb levels (all *p* < 0.05). Heart rate and SBP did not show significant univariate differences in the external validation cohort. Their retention in the final model was based on multivariable feature selection during model development, rather than on significance in the external cohort.

### 3.2 Feature selection and predictor identification

**Figure 2:**
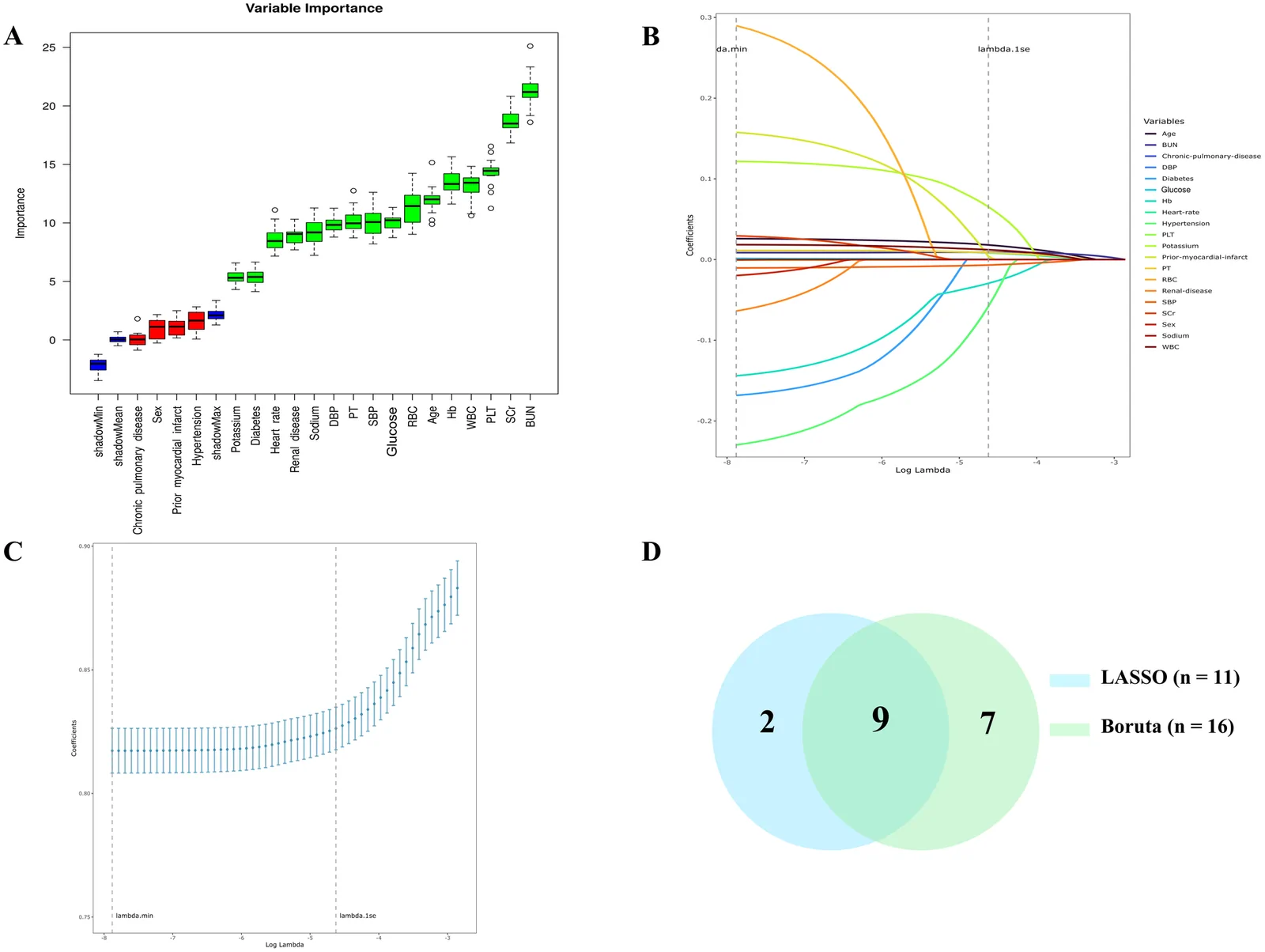
Feature selection and predictor identification. **(A)** Boruta-derived variable importance of the 21 initial candidate variables. **(B)** LASSO coefficient profiles of the candidate variables according to log(*λ*). **(C)** 10-fold cross-validation for selection of the optimal penalty parameter *λ* in the LASSO model. **(D)** Overlap between variables selected by Boruta and LASSO, yielding nine final predictors for model construction. SBP, systolic blood pressure; WBC, white blood cell count; PT, prothrombin time; BUN, blood urea nitrogen; Hb, hemoglobin; LASSO, least absolute shrinkage and selection operator.

A total of 21 initial candidate variables were included in the feature selection process. The Boruta algorithm selected 16 important variables for in-hospital mortality prediction, including age, diabetes, renal disease, SBP, DBP, heart rate, WBC, Hb, RBC, PLT, BUN, SCr, glucose, potassium, sodium, and PT (Figure 2A). LASSO regression was then applied for further variable selection. As the penalty parameter *λ* increased, the regression coefficients progressively shrank toward zero (Figure 2B). Using 10-fold cross-validation to determine the optimal penalty parameter (Figure 2C), LASSO retained 11 candidate variables. The intersection of the variables selected by Boruta and LASSO yielded nine final predictors for subsequent model construction: age, heart rate, SBP, WBC, PT, BUN, glucose, Hb, and potassium (Figure 2D).

### 3.3 Development and internal validation of machine learning models

**Figure 3:**
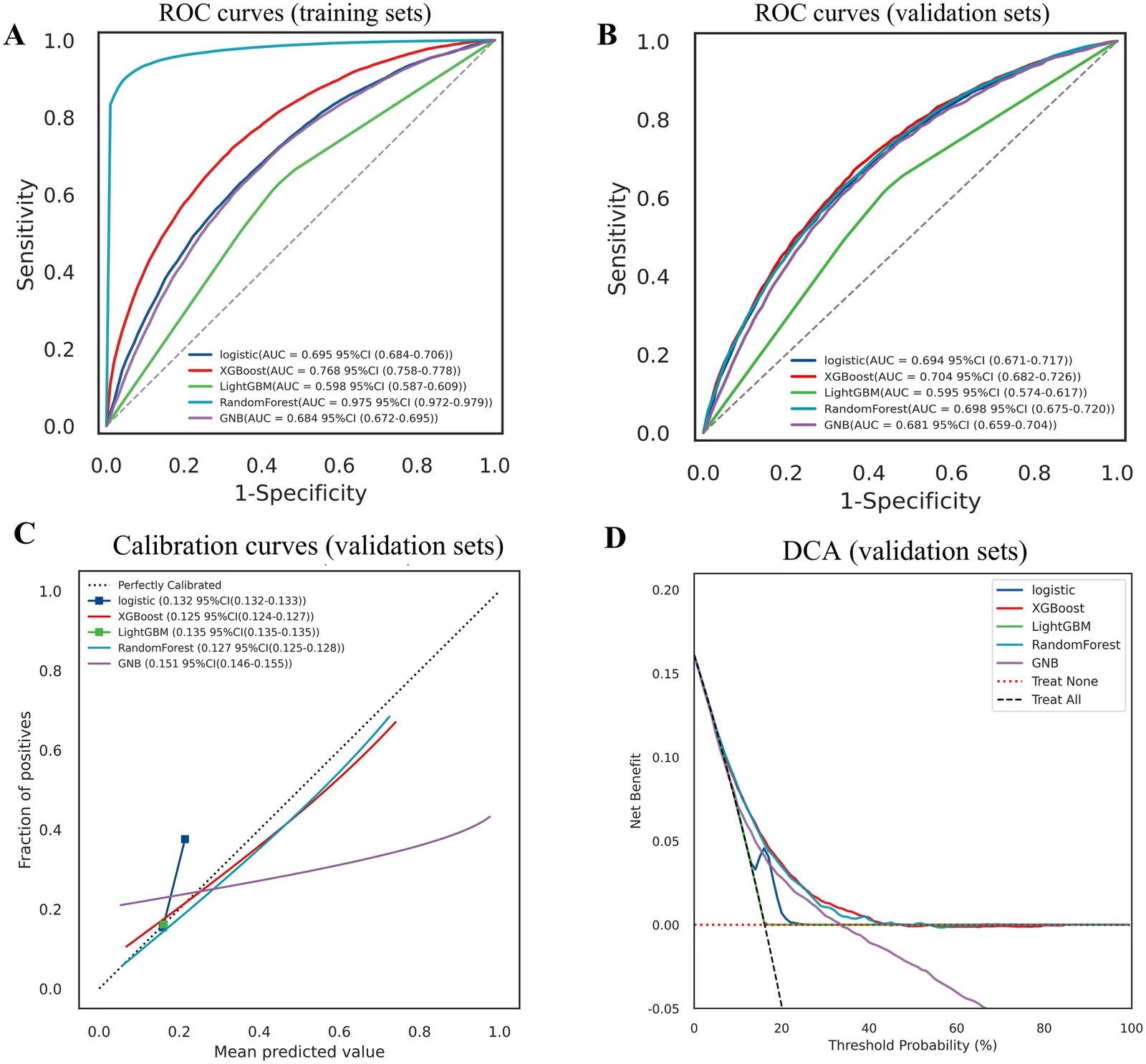
Internal validation performance of the candidate machine learning models. **(A)** ROC curves of the candidate models in the training sets; **(B)** ROC curves of the candidate models in the validation sets; **(C)** Calibration curves of the candidate models in the validation sets; **(D)** Decision curve analysis of the candidate models in the validation sets. LR, logistic regression; XGBoost, extreme gradient boosting; LightGBM, light gradient boosting machine; RF, random forest; GNB, Gaussian naïve Bayes; ROC, receiver operating characteristic; AUC, area under the receiver operating characteristic curve.

Based on the nine selected features, five machine learning models, including LR, XGBoost, LightGBM, RF, and GNB, were developed. A 5-fold nested CV framework combined with grid search was used for hyperparameter tuning and internal validation. The comparative performance of these models is summarized in Table 3 and Figure 3. Overall, XGBoost showed the most balanced performance across the outer-loop validation sets, whereas RF showed evidence of overfitting.

**Table 3:** Predictive performance of candidate machine learning models in the MIMIC-IV development cohort using nested 5-fold cross-validation.

Model	Dataset	AUC (95% CI)	Acc	Sens	Spec	PPV	NPV	F1 score
Logistic regression	CV–Train	0.695 (0.684–0.706)	0.638	0.652	0.635	0.256	0.905	0.367
	CV–Val	0.694 (0.671–0.717)	0.637	0.646	0.635	0.255	0.904	0.365
XGBoost	CV–Train	0.768 (0.758–0.778)	0.694	0.706	0.692	0.306	0.925	0.427
	CV–Val	0.704 (0.682–0.726)	0.665	0.624	0.673	0.268	0.903	0.375
LightGBM	CV–Train	0.598 (0.587–0.609)	0.574	0.631	0.563	0.218	0.889	0.323
	CV–Val	0.595 (0.574–0.617)	0.573	0.626	0.563	0.217	0.887	0.321
Random forest	CV–Train	0.975 (0.972–0.979)	0.967	0.810	0.998	0.985	0.965	0.889
	CV–Val	0.698 (0.675–0.720)	0.838	0.051	0.990	0.497	0.844	0.090
GNB	CV–Train	0.684 (0.672–0.695)	0.610	0.684	0.596	0.246	0.908	0.361
	CV–Val	0.681 (0.659–0.704)	0.608	0.676	0.595	0.243	0.906	0.357

CV, cross-validation; CV–Train, model performance in the training sets during nested cross-validation; CV–Val, model performance in the validation sets during nested cross-validation; AUC, area under the receiver operating characteristic curve; CI, confidence interval; Acc, accuracy; Sens, sensitivity; Spec, specificity; PPV, positive predictive value; NPV, negative predictive value.

During nested CV, the RF model achieved the highest AUC in the training sets, at 0.975 (95% CI 0.972–0.979), whereas its AUC in the outer-loop validation sets was 0.698 (95% CI 0.675–0.720) (Figures 3A,B). The absolute difference in AUC between the training and validation sets was 0.277. In contrast, XGBoost achieved the highest outer-loop validation AUC, at 0.704 (95% CI 0.682–0.726). The validation AUCs of the RF, LR, GNB, and LightGBM models were 0.698 (95% CI 0.675–0.720), 0.694 (95% CI 0.671–0.717), 0.681 (95% CI 0.659–0.704), and 0.595 (95% CI 0.574–0.617), respectively (Figure 3B).

Based on the pooled out-of-fold predictions from the outer loop, the calibration curve showed acceptable agreement between the predicted probabilities and observed outcomes for XGBoost, with a Brier score of 0.125 (95% CI 0.124–0.127), a calibration slope of 1.146, and a CITL of 0.004 in the validation sets (Figure 3C). DCA based on the same outer-loop validation predictions showed that XGBoost provided a higher net benefit than the other candidate models across a wide range of threshold probabilities in the development cohort (Figure 3D). Taking into account the outer-loop validation AUC, classification metrics, calibration performance, overall prediction error, and DCA results, XGBoost demonstrated the most balanced overall performance among the candidate models and was therefore selected as the final modeling algorithm.

The selected XGBoost model was further tuned. The final model used a binary logistic objective function, with the learning rate, max depth, min child weight, and L2 regularization coefficient set to 0.05, 3, 5, and 2, respectively. After hyperparameter refinement, the finalized XGBoost model achieved an AUC of 0.712 (95% CI 0.690–0.734), with generally stable classification performance (Supplementary Table S5).

### 3.4 External validation of the final model

**Figure 4:**
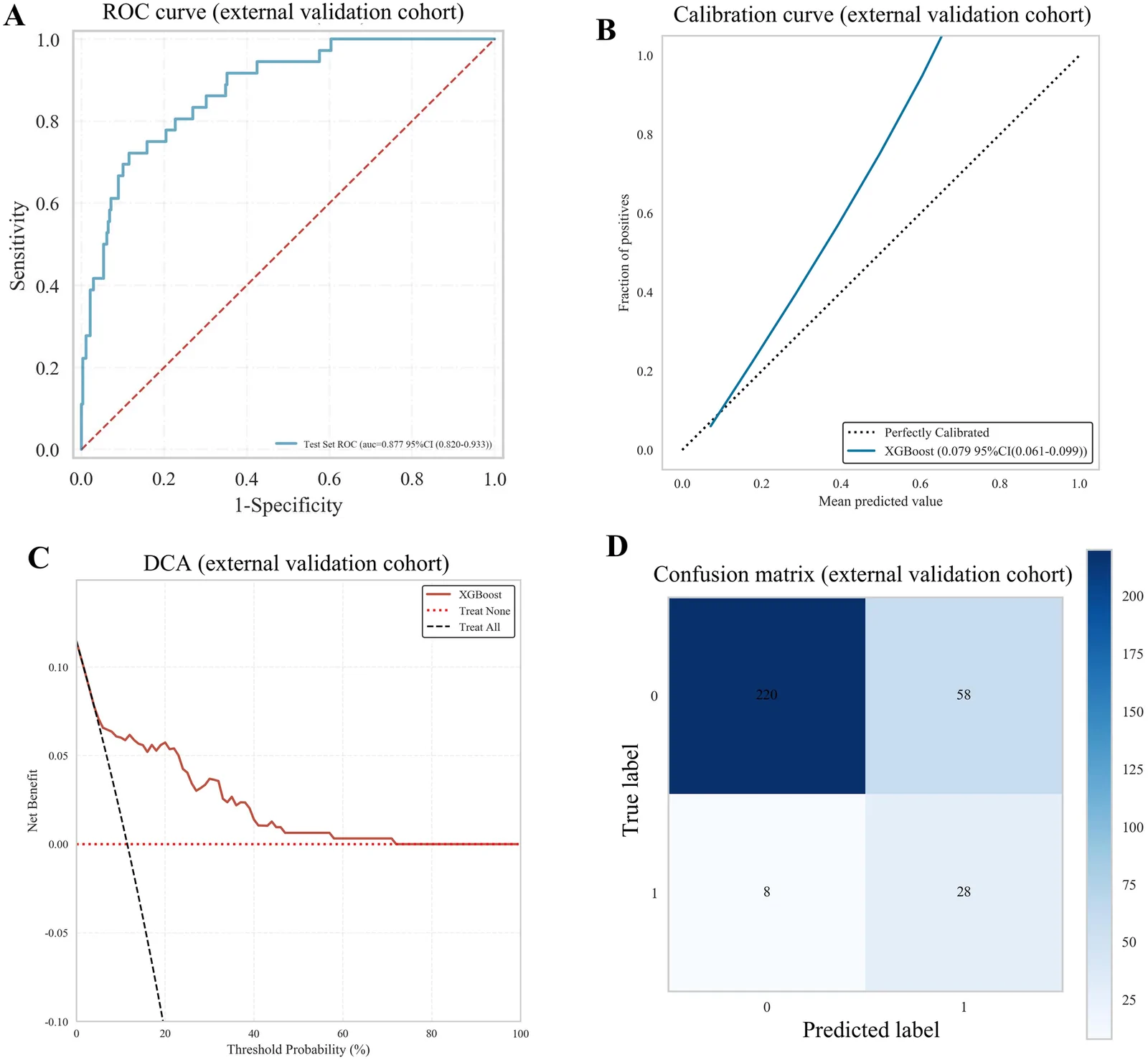
**External validation performance of the final XGBoost model. (A)** ROC curve of the final XGBoost model in the external validation cohort; **(B)** Calibration curve of the final XGBoost model in the external validation cohort; **(C)** Decision curve analysis of the final XGBoost model in the external validation cohort; **(D)** Confusion matrix of the final XGBoost model in the external validation cohort. XGBoost, extreme gradient boosting; ROC, receiver operating characteristic; AUC, area under the receiver operating characteristic curve; DCA, decision curve analysis.

In the independent external validation cohort, the final model showed favorable discrimination and acceptable calibration, although these external findings should be interpreted cautiously given the limited number of events. Specifically, the final XGBoost model achieved an AUC of 0.877 (95% CI 0.820–0.933) (Figure 4A), and the grouped calibration plot suggested acceptable agreement between predicted and observed probabilities, with a Brier score of 0.079 (95% CI 0.061–0.099) (Figure 4B). At the prespecified probability cutoff of 0.157, derived from the development cohort using the maximum Youden index, the accuracy, sensitivity, specificity, PPV, NPV, and F1 score were 0.790, 0.778, 0.791, 0.326, 0.965, and 0.459, respectively (Supplementary Table S6). DCA showed that the model yielded a higher net benefit than the treat-all and treat-none strategies over a wide range of threshold probabilities in the external validation cohort, with the net benefit approaching zero at around 70% (Figure 4C). The confusion matrix showed that the model correctly classified 220 in-hospital survivors and 28 in-hospital nonsurvivors, whereas 58 survivors were misclassified as high risk and 8 nonsurvivors were misclassified as low risk (Figure 4D). To further explore the unexpectedly higher discrimination observed in the external validation cohort, we additionally compared the distributions of the nine final predictors and in-hospital all-cause mortality between the development and external validation cohorts (Supplementary Table S7). Several predictors showed noticeable between-cohort differences, particularly SBP, PT, Hb, WBC, and heart rate, whereas BUN was relatively similar between cohorts. To further contextualize threshold-dependent classification performance, Supplementary Table S8 summarizes the sensitivity-specificity trade-off of the finalized XGBoost model in the development cohort under alternative threshold selection strategies.

### 3.5 Comparison with established ICU severity scores

As shown in Supplementary Figure S1A, the AUCs of SOFA and APS III were 0.761 and 0.754, respectively, in the development cohort.

We further compared the clinical net benefit of the final model with those of SOFA and APS III by DCA. As shown in Supplementary Figure S1B, the final model, SOFA, and APS III all provided net benefit over the treat-all and treat-none strategies across parts of the evaluated threshold range, although the magnitude and range of net benefit differed among them.

### 3.6 Model interpretability and nonlinear association analysis

**Figure 5:**
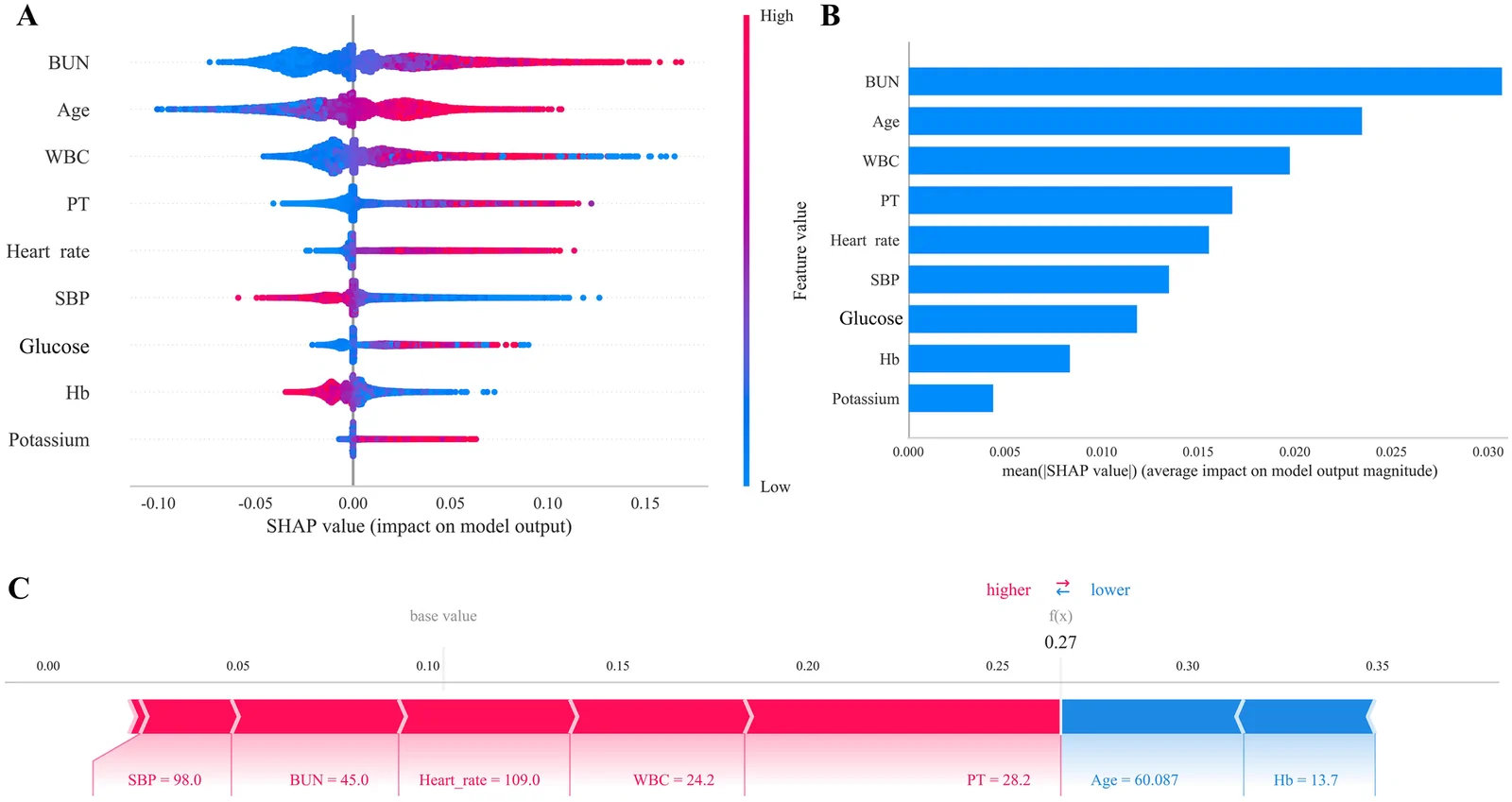
SHAP-based interpretation of the final XGBoost model. **(A)** SHAP summary plot (beeswarm) of the nine predictors; **(B)** Feature importance plot based on mean absolute SHAP values; **(C)** SHAP force plot for an illustrative patient. XGBoost, extreme gradient boosting; SHAP, Shapley additive explanations; BUN, blood urea nitrogen; WBC, white blood cell count; PT, prothrombin time; SBP, systolic blood pressure; Hb, hemoglobin.

SHAP interpretation of the final XGBoost model was performed in the development cohort using shap.TreeExplainer (TreeSHAP). The SHAP summary plot indicated that BUN made the largest contribution to model output, followed by age, WBC, PT, heart rate, SBP, glucose, Hb, and potassium. The feature importance bar plot showed a consistent ranking of these predictors (Figures 5A,B).

In terms of directionality, higher BUN, older age, higher WBC, longer PT, higher heart rate, and higher glucose were generally associated with higher SHAP values, indicating an increased predicted risk of in-hospital mortality. Lower SBP and lower Hb were also generally associated with a higher predicted risk, whereas potassium contributed relatively little overall. At the individual level, the SHAP force plot showed that, for an illustrative survivor from the development cohort selected to demonstrate individual-level model interpretation, SBP, BUN, heart rate, WBC, and PT were the main factors increasing the predicted risk, whereas younger age and higher Hb partly offset that risk (Figure 5C). The expected value used in the individual force plot was 0.104.

As a complementary analysis, RCS analyses were performed for the continuous variables included in the final model in the development cohort. All continuous predictors showed significant overall associations with in-hospital mortality (all *P* for overall <0.001). Significant nonlinearity was observed for BUN, WBC, PT, heart rate, SBP, and glucose (all *P* for nonlinearity <0.001), whereas age, Hb, and potassium did not show statistically significant nonlinearity. The detailed spline curves are presented in Supplementary Figure S2.

### 3.7 Construction of the online prediction tool

**Figure 6:**
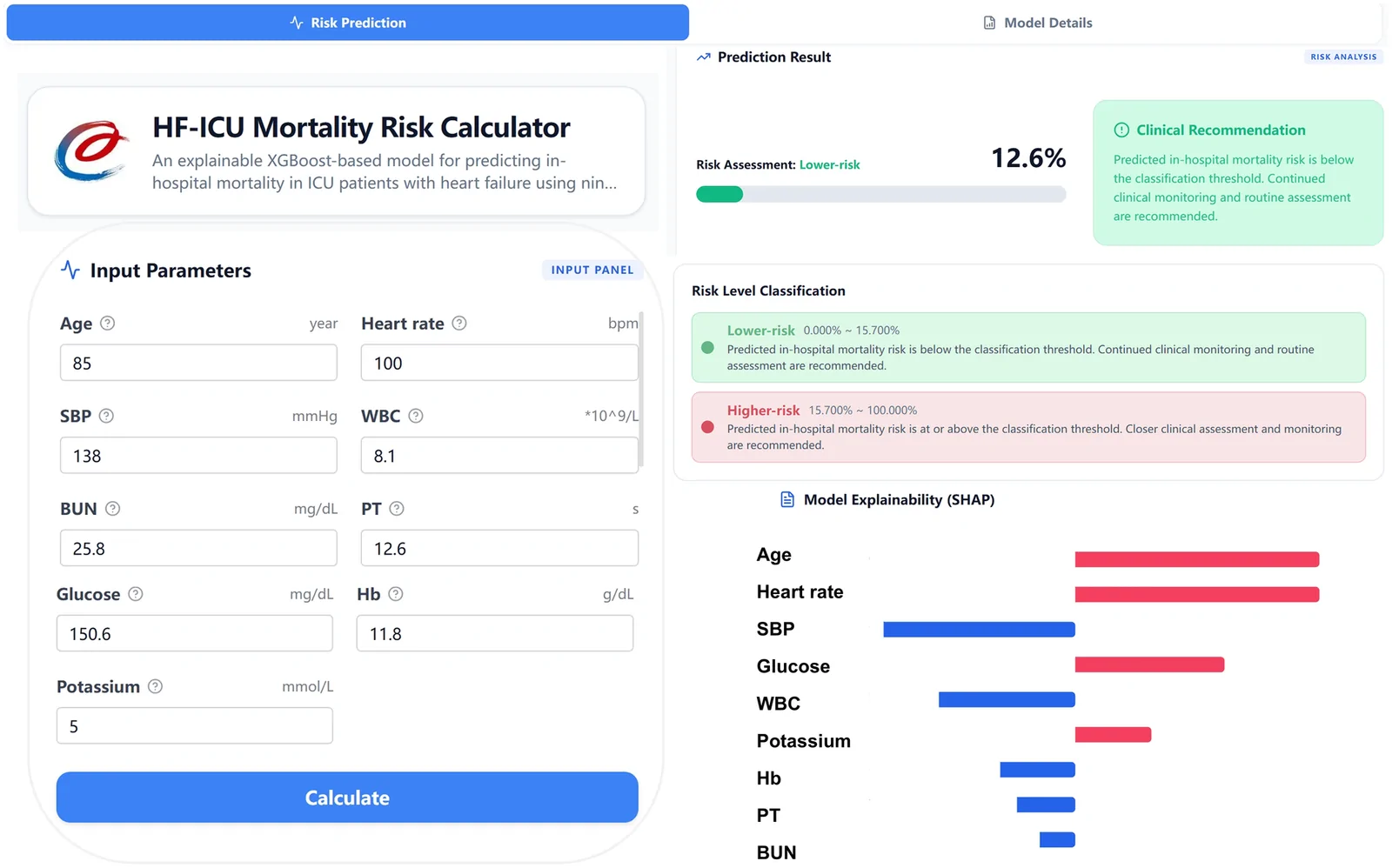
Web-based interface of the online prediction tool derived from the final XGBoost model.

Based on the final XGBoost model, a web-based risk prediction tool was developed (Figure 6) to facilitate bedside use and individualized risk assessment. The tool incorporated the nine routinely available clinical variables included in the final model and provided the predicted probability of in-hospital mortality, the corresponding risk classification, and individualized SHAP-based interpretive information. The web address of the online tool is provided in Supplementary Appendix S1.

## 4 Discussion

In this study, we developed and externally validated an explainable machine learning model to predict in-hospital all-cause mortality among ICU patients with HF. Among the candidate algorithms, XGBoost demonstrated the most balanced overall performance during model development and showed encouraging external discrimination and overall prediction error in an independent validation cohort, although the external findings require cautious interpretation because of cohort differences and limited event counts. The final model incorporated only nine routinely available variables obtained early after ICU admission, and SHAP analysis further identified BUN, age, and WBC as the most influential predictors. In addition, we developed an online prediction tool to facilitate bedside use. Collectively, these findings suggest that an interpretable machine learning model based on routinely available early clinical data may provide useful support for early risk stratification in critically ill patients with HF.

Previous studies have applied ML algorithms to predict adverse outcomes in patients with HF, including in-hospital mortality, short-term mortality, and readmission, and have generally reported promising performance (3, 7, 13, 14). Building on this literature, recent studies have increasingly examined ICU or otherwise high-risk HF cohorts and incorporated SHAP or related explainable machine learning methods into models derived from public critical-care databases such as MIMIC-IV and the eICU Collaborative Research Database (eICU-CRD). Although these studies have generally shown reasonable discriminative performance, many still face practical barriers to clinical implementation, including reliance on relatively large or setting-specific feature panels, limited simplification for bedside use, and, in some cases, the absence of fully independent external real-world validation (18–20). In this context, the present study contributes to the literature in several ways. First, the final model used a relatively parsimonious set of nine predictors, which may enhance its practical applicability in ICU settings. Second, multiple candidate algorithms were compared within a nested CV framework, and XGBoost was selected based on the most balanced overall performance rather than training performance alone. Third, the model was further evaluated in an independent external cohort, thereby increasing confidence in its robustness and potential generalizability. Fourth, SHAP-based interpretation was integrated with an online prediction tool to facilitate practical bedside use. We also benchmarked the proposed model against two established ICU severity scores, SOFA and APS III, in the development cohort to better contextualize its performance. These analyses showed that conventional ICU severity scores remained competitive in this setting. Therefore, the proposed model may be better interpreted as a supplementary HF-specific risk-stratification tool rather than a replacement for established ICU severity scores. Together, these features distinguish the present study from many previously developed prediction models in similar clinical settings, including our own prior work on prognostic biomarkers in HF. Our previous study examined the prognostic association of the CHG index in HF, whereas the present study aimed to develop and validate an explainable multivariable model for early mortality prediction in ICU patients with HF.

The present findings also provide insight into the comparative performance of different algorithms in this setting. RF appeared prone to overfitting, whereas XGBoost showed the most robust overall validation performance. This pattern is clinically understandable because ICU patients with HF represent a heterogeneous population in whom mortality risk is likely shaped by nonlinear relationships and higher-order interactions among hemodynamic, renal, inflammatory, coagulation, and metabolic variables (4, 5, 9–11). In such settings, boosting-based approaches may offer advantages over simpler linear models, as well as over more flexible models that are particularly susceptible to overfitting (7, 13). Notably, the favorable performance of XGBoost in our study was achieved without requiring an excessive number of predictors, further supporting the feasibility of this modeling strategy in routine practice.

In the independent external validation cohort, the final model showed good discrimination and acceptable calibration, and DCA suggested potential clinical utility across a range of threshold probabilities. Because the clinical implications of acting on model predictions may vary across care settings, the prespecified cutoff of 0.157 should not be regarded as the only clinically meaningful decision threshold. Instead, DCA provides a complementary assessment of potential clinical utility across a range of threshold probabilities. From a practical perspective, the high NPV observed in the external validation cohort suggests that the model may be particularly useful for identifying patients at relatively low risk of in-hospital mortality, thereby helping to inform early triage, monitoring decisions, and ICU resource allocation. By contrast, the PPV was relatively limited, indicating that patients classified as high risk should not be assumed to have a poor outcome with certainty, but rather should undergo closer assessment and monitoring. This finding underscores the need for cautious interpretation of high-risk predictions in clinical practice. It is also noteworthy that the AUC in the external validation cohort was higher than that in the internal validation sets. However, this finding should not be interpreted as evidence of superior external performance. In prediction-model validation, a higher external AUC may arise when the validation cohort differs from the development cohort in case mix, predictor distributions, or outcome frequency, thereby making outcome separation easier without necessarily indicating better transportability or generalizability (38, 39). In our study, clear between-cohort differences in several predictors and in outcome frequency may have contributed to the apparently stronger discrimination observed in the external cohort. Therefore, the external validation findings should be regarded as supportive but provisional, rather than as definitive evidence of superior model performance or broad clinical applicability.

The predictor profile identified by the final model is also clinically meaningful. Among the nine retained variables, BUN, age, and WBC contributed most strongly to the model's predictions. Elevated BUN may reflect not only impaired renal function but also reduced renal perfusion, venous congestion, and catabolic stress, and thus may serve as an integrated marker of cardiorenal dysfunction and disease severity in critically ill patients with HF (19, 40, 41). Age is a well-established prognostic factor and likely captures reduced physiologic reserve, multimorbidity burden, frailty, and diminished tolerance to acute hemodynamic stress and intensive treatment (14, 42, 43). WBC may reflect inflammatory activation, infectious burden, stress response, or immune dysregulation, all of which may contribute to clinical deterioration in HF (10, 44, 45). The remaining predictors, including PT, heart rate, SBP, glucose, Hb, and potassium, reflected additional dimensions of coagulation status, circulatory and hemodynamic instability, metabolic stress, oxygen-carrying capacity, and internal homeostasis (9–11, 46, 47). Taken together, these variables suggest that short-term mortality in ICU patients with HF is influenced by multiple interrelated processes, particularly cardiorenal dysfunction, systemic inflammation, hemodynamic instability, and metabolic disturbance.

An important strength of the present study is the incorporation of model interpretability. Although machine learning models may improve predictive performance, limited transparency remains a major barrier to clinical adoption. In this study, SHAP analysis was used to characterize both the global importance of predictors and their contributions to individual predictions. Overall, the SHAP findings were clinically coherent and indicated that mortality risk was driven by a combination of renal dysfunction, aging, inflammation, coagulation abnormality, hemodynamic instability, and metabolic disturbance. These findings were broadly consistent with clinical expectations (4, 11, 23, 24). In addition, the supplementary RCS analyses supported significant nonlinear associations for several continuous predictors, including BUN, WBC, PT, heart rate, SBP, and glucose, indicating that the relationships between these variables and mortality risk may not be adequately captured by simple linear assumptions alone. At the individual level, SHAP further illustrated that elevated risk may arise from different combinations of abnormal features across patients. This layered interpretive framework may improve transparency, facilitate clinician understanding of model outputs, and enhance confidence in the model's clinical use.

From an implementation perspective, the model has several practical advantages. All nine predictors are routinely available early after ICU admission and do not require specialized testing or complex score calculation. The model was externally validated and subsequently translated into an online prediction tool that provides both risk estimates and individualized SHAP-based interpretive information. This combination may improve accessibility at the bedside and help bridge the gap between predictive analytics and clinical decision support. Nevertheless, the tool should be viewed as a means of supporting clinician judgment rather than replacing it. For patients identified as high risk, comprehensive evaluation incorporating etiology, treatment response, imaging findings, and dynamic clinical changes remains essential (48, 49).

Several limitations should be acknowledged. First, this was a retrospective observational study and was therefore subject to potential selection bias, information bias, and residual confounding. Second, although an independent external validation cohort was included, it was derived from a single center and had a relatively limited sample size and number of outcome events. Differences in case mix and mortality rates were also observed between the development and external validation cohorts. In addition, some between-cohort differences in baseline comorbidities may have been influenced by differences in data sources and comorbidity ascertainment, which further limits direct comparability. Nevertheless, the preservation of favorable discrimination in this heterogeneous external cohort provides only preliminary support for model robustness, not definitive evidence of broad transportability. As a result, the external validation results, especially the calibration-related findings, should be considered provisional and interpreted cautiously. Further large, diverse, and preferably prospective multicenter external validation is warranted before broader clinical implementation. Third, the model was developed using static variables collected early after ICU admission and did not incorporate dynamic physiologic trends, treatment-related variables, echocardiographic parameters, or more detailed etiologic information, all of which may provide additional prognostic value. Fourth, feature selection was conducted in the development cohort before nested cross-validation, which may have introduced some degree of optimistic bias. In addition, after XGBoost had been selected as the final algorithm, its hyperparameters were further refined within the development cohort outside the outer loop of nested cross-validation. Therefore, the performance summary of the finalized model should be interpreted as potentially optimistic relative to the primary nested cross-validation estimate used for comparison among candidate algorithms. Nevertheless, the model was further assessed in an independent external validation cohort, providing a more rigorous evaluation of generalizability. Fifth, dietary intake, baseline nutritional status, and nutritional interventions during hospitalization were not systematically captured in the available datasets, precluding an assessment of their potential influence on in-hospital all-cause mortality risk and model performance (50). Future prospective studies should incorporate detailed nutritional assessment and longitudinal monitoring of these factors to better define the role of nutrition in risk stratification and clinical outcomes.

## 5 Conclusion

We developed and externally validated an explainable XGBoost-based model for predicting in-hospital all-cause mortality in ICU patients with HF. The model incorporated nine routinely available variables obtained early after ICU admission, demonstrated good discrimination and acceptable calibration, and provided interpretable predictions through SHAP analysis. Together with the accompanying online prediction tool, it may offer practical supplementary support for early risk stratification and individualized risk assessment in critically ill patients with HF. Further prospective multicenter validation is warranted to confirm generalizability and clinical utility.

## Data Availability

The raw data supporting the conclusions of this article will be made available by the authors, without undue reservation.
