# Supplementary material for: Development and external validation of an explainable machine learning model for in-hospital mortality risk stratification in intensive care unit patients with heart failure
Source: Front Cardiovasc Med. 2026 Sep 4;13:1942393. doi: 10.3389/fcvm.2026.1942393 (PMC13585971; doi:10.3389/fcvm.2026.1942393)
Supplement: Supplementary file 1 [file Datasheet1.pdf]

TRIPOD+AI checklist

Section/topic	Item	Development /evaluation*	Checklist item	Reported on page and line numbers
Title				
Title	1	D;E	Identify the study as developing or evaluating the performance of a multivariable prediction model, the target population, and the outcome to be predicted	Page 1, lines 1–3.
Abstract				
Abstract	2	D;E	See TRIPOD+AI for Abstracts checklist	Page 2, lines 41–69. Structured abstract reporting background, objectives, data source, outcome, model type and validation approach, performance measures, main results, and conclusion. Registration information was not reported in the abstract.
Introduction				
Background	3a	D;E	Explain the healthcare context (including whether diagnostic or prognostic) and rationale for developing or evaluating the prediction model, including references to existing models	Pages 3–4, lines 84–138.
	3b	D;E	Describe the target population and the intended purpose of the prediction model in the context of the care pathway, including its intended users (eg, healthcare professionals, patients, public)	Page 4, lines 131–138.

	3c	D;E	Describe any known health inequalities between sociodemographic groups	Not reported. No prespecified assessment of health inequalities across relevant sociodemographic groups was described.
Objectives	4	D;E	Specify the study objectives, including whether the study describes the development or validation of a prediction model (or both)	Page 4, lines 131–138.
Methods				
Data	5a	D;E	Describe the sources of data separately for the development and evaluation datasets (eg, randomised trial, cohort, routine care or registry data), the rationale for using these data, and representativeness of the data	Pages 4–5, lines 141–169.
	5b	D;E	Specify the dates of the collected participant data, including start and end of participant accrual; and, if applicable, end of follow-up	Page 4, lines 149–165; Page 5, lines 167–169.
Participants	6a	D;E	Specify key elements of the study setting (eg, primary care, secondary care, general population) including the number and location of centres	Pages 4–5, lines 149–169; Pages 5–6, lines 171–213.
	6b	D;E	Describe the eligibility criteria for study participants	Page 5, lines 176–190.
	6c	D;E	Give details of any treatments received, and how they were handled during model	Not applicable. Treatment variables were not included as candidate predictors,

			development or evaluation, if relevant	and treatment use was not part of model development or validation.
Data preparation	7	D;E	Describe any data pre-processing and quality checking, including whether this was similar across relevant sociodemographic groups	Pages 5–6, lines 206–239.
Outcome	8a	D;E	Clearly define the outcome that is being predicted and the time horizon, including how and when assessed, the rationale for choosing this outcome, and whether the method of outcome assessment is consistent across sociodemographic groups	Page 4, lines 131–138; Page 5, lines 192–196.
	8b	D;E	If outcome assessment requires subjective interpretation, describe the qualifications and demographic characteristics of the outcome assessors	Not applicable. Outcome determination was based on retrospective clinical record data and did not involve subjective outcome adjudication procedures.
	8c	D;E	Report any actions to blind assessment of the outcome to be predicted	Not applicable. As this was a retrospective study using existing records, blinding of outcome assessment was not undertaken.
Predictors	9a	D	Describe the choice of initial predictors (eg, literature, previous models, all available predictors) and any pre-selection of predictors before model building	Page 5, lines 197–205; Pages 6–7, lines 230–239.
	9b	D;E	Clearly define all	Page 5, lines 197–213;

			predictors, including how and when they were measured (and any actions to blind assessment of predictors for the outcome and other predictors)	Page 6, lines 225–229.
	9c	D;E	If predictor measurement requires subjective interpretation, describe the qualifications and demographic characteristics of the predictor assessors	Not applicable. Predictor assessment was based on routinely collected objective variables and did not involve subjective interpretation requiring blinding.
Sample size	10	D;E	Explain how the study size was arrived at (separately for development and evaluation), and justify that the study size was sufficient to answer the research question. Include details of any sample size calculation	Not reported as a formal sample size calculation. Cohort sizes are reported on Page 8, lines 320–322; limitations related to sample size and external validation event counts are discussed on Page 14, lines 560–589.
Missing data	11	D;E	Describe how missing data were handled. Provide reasons for omitting any data	Page 6, lines 215–229.
Analytical methods	12a	D	Describe how the data were used (eg, for development and evaluation of model performance) in the analysis, including whether the data were partitioned, considering any sample size requirements	Pages 6–8, lines 240–317.
	12b	D	Depending on the type of model, describe how predictors were handled in the analyses	Partly reported: Page 5, lines 206–213; Page 6, lines 215–229. Predictor extraction

			(functional form, rescaling, transformation, or any standardisation)	window, variable processing, and missing-data handling are described, but full details of all preprocessing and transformation steps are not reported.
	12c	D	Specify the type of model, rationale†, all model building steps, including any hyperparameter tuning, and method for internal validation	Pages 6–8, lines 240–317; Pages 9–10, lines 358–386.
	12d	D;E	Describe if and how any heterogeneity in estimates of model parameter values and model performance was handled and quantified across clusters (eg, hospitals, countries). See TRIPOD-Cluster for additional considerations‡	Not reported as a formal heterogeneity analysis. Differences between development and external validation cohorts are described, but cluster-level or site-level heterogeneity in model performance was not formally examined.
	12e	D;E	Specify all measures and plots used (and their rationale) to evaluate model performance (eg, discrimination, calibration, clinical utility) and, if relevant, to compare multiple models	Pages 7–8, lines 279–317; Pages 9–11, lines 372–446.
	12f	E	Describe any model updating (eg, recalibration) arising from the model evaluation, either overall or for particular sociodemographic groups or settings	Not applicable. No model updating or recalibration was performed following external validation.
	12g	E	For model evaluation, describe how the model	Page 8, lines 315–317; Page 10, lines 388–

			predictions were calculated (eg, formula, code, object, application programming interface)	396; Page 11, lines 440–446.
Class imbalance	13	D;E	If class imbalance methods were used, state why and how this was done, and any subsequent methods to recalibrate the model or the model predictions	Page 7, lines 257–260.
Fairness	14	D;E	Describe any approaches that were used to address model fairness and their rationale	Not reported. No formal fairness analysis or subgroup performance assessment across sociodemographic groups was presented.
Model output	15	D	Specify the output of the prediction model (eg, probabilities, classification). Provide details and rationale for any classification and how the thresholds were identified	Pages 7–8, lines 298–304; Page 10, lines 394–396.
Training versus evaluation	16	D;E	Identify any differences between the development and evaluation data in healthcare setting, eligibility criteria, outcome, and predictors	Pages 4–6, lines 149–213; Page 8, lines 320–334; Table 1 (Page 21).
Ethical approval	17	D;E	Name the institutional research board or ethics committee that approved the study and describe the participant informed consent or the ethics committee waiver of informed consent	Pages 4–5, lines 143–169; Page 15, lines 623–629.
Open science				
Funding	18a	D;E	Give the source of funding and the role of the funders for the	Page 15, lines 607–609. Funding sources are reported; the

			present study	specific role of the funders was not explicitly described.
Conflicts of interest	18b	D;E	Declare any conflicts of interest and financial disclosures for all authors	Page 15, lines 610–611.
Protocol	18c	D;E	Indicate where the study protocol can be accessed or state that a protocol was not prepared	Not reported. No protocol document or protocol access information was provided.
Registration	18d	D;E	Provide registration information for the study, including register name and registration number, or state that the study was not registered	Not reported. No registration number, registry name, or repository identifier was provided.
Data sharing	18e	D;E	Provide details of the availability of the study data	Page 15, lines 618–622.
Code sharing	18f	D;E	Provide details of the availability of the analytical code§	Partly reported: Page 8, lines 313–317; Page 11, lines 440–446. The manuscript states that the final trained model was retained in serialized format and deployed through an online calculator, but no public code repository or full analytical pipeline is provided.
Patient and public involvement				
Patient and public involvement	19	D;E	Provide details of any patient and public involvement during the design, conduct, reporting, interpretation, or dissemination of the study or state no involvement	Not reported. No patient or public involvement in study design, conduct, reporting, interpretation, or dissemination was described.

Result				
Participants	20a	D;E	Describe the flow of participants through the study, including the number of participants with and without the outcome and, if applicable, a summary of the follow-up time. A diagram may be helpful	Page 5, lines 176–191; Page 8, lines 320–322; Figure 1.
	20b	D;E	Report the characteristics overall and, where applicable, for each data source or setting, including the key dates, key predictors (including demographics), treatments received, sample size, number of outcome events, follow-up time, and amount of missing data. A table may be helpful. Report any differences across key demographic groups	Page 5, lines 176–191; Page 8, lines 320–322; Figure 1. Supplementary Tables S2 and S4
	20c	E	For model evaluation, show a comparison with the development data of the distribution of important predictors (demographics, predictors, and outcome)	Table 1 (Page 21); Page 10, lines 402–409; Supplementary Table S7.
Model development	21	D;E	Specify the number of participants and outcome events in each analysis (eg, for model development, hyperparameter tuning, model evaluation)	Page 7, lines 261–270; Page 8, lines 320–322; Pages 10–11, lines 384–396. Separate event counts for each internal validation fold were not reported.
Model specification	22	D	Provide details of the full prediction model (eg, formula, code, object, application programming interface) to allow	Partly reported: Page 8, lines 315–317; Page 10, lines 382–386; Pages 10–11, lines 394–396; Page 11,

			predictions in new individuals and to enable third party evaluation and implementation, including any restrictions to access or reuse (eg, freely available, proprietary)¶	lines 440–446. The final model type, retained predictors, decision threshold, and online calculator are described, but full model code, API details, and reuse conditions are not fully reported.
Model performance	23a	D;E	Report model performance estimates with confidence intervals, including for any key subgroups (eg, sociodemographic). Consider plots to aid presentation	Table 3 (Page 23); Pages 9–11, lines 358–446; Figures 3–4; Supplementary Tables S5–S6 and Supplementary Figure S1. No subgroup-specific performance estimates were reported.
	23b	D;E	If examined, report results of any heterogeneity in model performance across clusters. See TRIPOD-Cluster for additional details‡	Not applicable. Heterogeneity in model performance across clusters or settings was not formally assessed.
Model updating	24	E	Report the results from any model updating, including the updated model and subsequent performance	Not applicable. No model updating or recalibration after external validation was undertaken.
Discussion				
Interpretation	25	D;E	Give an overall interpretation of the main results, including issues of fairness in the context of the objectives and previous studies	Pages 11–14, lines 448–589. No formal fairness or subgroup disparity analyses were reported.
Limitations	26	D;E	Discuss any limitations of the study (such as a non-representative sample, sample size, overfitting, missing data)	Page 14, lines 560–589.

			and their effects on any biases, statistical uncertainty, and generalisability	
Usability of the model in the context of current care	27a	D	Describe how poor quality or unavailable input data (eg, predictor values) should be assessed and handled when implementing the prediction model	Partly reported: Page 5, lines 206–213; Page 6, lines 215–229; Page 14, lines 550–559. Development-stage preprocessing and missing-data handling are described, but no detailed operational protocol is provided for low-quality, missing, or unavailable inputs during real-world implementation.
	27b	D	Specify whether users will be required to interact in the handling of the input data or use of the model, and what level of expertise is required of users	Page 4, lines 137–138; Page 11, lines 440–446; Page 14, lines 550–559.
	27c	D;E	Discuss any next steps for future research, with a specific view to applicability and generalisability of the model	Page 14, lines 571–589.
