# Supplementary material for: Development and external validation of an explainable machine learning model for in-hospital mortality risk stratification in intensive care unit patients with heart failure
Source: Front Cardiovasc Med. 2026 Sep 4;13:1942393. doi: 10.3389/fcvm.2026.1942393 (PMC13585971; doi:10.3389/fcvm.2026.1942393)
Supplement: Supplementary file 2 [file Supplementaryfile1.docx]

**Supplementary materials**

Supplementary Table S1: **Unit conversion factors for laboratory variables.**

Supplementary Table S2: **Missingness of candidate continuous variables in the MIMIC-IV development cohort.**

Supplementary Table S3: **Prespecified hyperparameter search space for candidate machine learning algorithms.**

Supplementary Table S4: **Comparison of the nine selected predictors between survivors and non-survivors in the external validation cohort.**

Supplementary Table S5: **Performance of the finalized XGBoost model in the MIMIC-IV development cohort after hyperparameter refinement.**

Supplementary Table S6: **External validation performance metrics of the final XGBoost model.**

Supplementary Table S7: **Cross-cohort comparison of the nine final predictors and in-hospital mortality between the development cohort and the external validation cohort.**

Supplementary Table S8: **Sensitivity and specificity under alternative threshold selection strategies for the finalized XGBoost model in the development cohort.**

Supplementary Figure S1: **Benchmark comparison with established ICU severity scores in the development cohort.** (A) ROC curves of SOFA and APS III for predicting in-hospital all-cause mortality; (B) DCA curve comparing the net benefit of the final XGBoost model, SOFA, APS III, and the treat-all and treat-none strategies across threshold probabilities.
Abbreviations: SOFA, Sequential Organ Failure Assessment; APS III, Acute Physiology Score III; ROC, receiver operating characteristic; AUC, area under the receiver operating characteristic curve; DCA, decision curve analysis.

Supplementary Figure S2. **Restricted cubic spline analyses of the nine continuous predictors retained in the final model in relation to in-hospital mortality in the development cohort.** All spline terms were fitted using 4 knots placed at the 5th, 35th, 65th, and 95th percentiles, corresponding to 3 degrees of freedom. **Abbreviations**: BUN, blood urea nitrogen; WBC, white blood cell count; PT, prothrombin time; SBP, systolic blood pressure; Hb, hemoglobin; CI, confidence interval.

**Supplementary Appendix S1**: **Web-based risk prediction tool**

A web-based calculator developed from the final XGBoost model is available for individualized prediction of in-hospital mortality in patients with heart failure. Users can input the nine predictors retained in the final model to obtain the predicted risk.

**Web address**: <https://next.xsmartanalysis.com/predictive/preview/100>

Table S1. **Unit conversion factors for laboratory variables.**

| **Variables** | **Original unit** | **Target unit** | **Conversion factor** | **Conversion formula** |
| --- | --- | --- | --- | --- |
| Glucose | mmol/L | mg/dL | 18.02 | mg/dL = mmol/L × 18.02 |
| Urea (converted to BUN) | Urea, mmol/L | BUN, mg/dL | 2.801 | BUN (mg/dL) = Urea (mmol/L) × 2.801 |
| SCr | μmol/L | mg/dL | 0.01131 | mg/dL = μmol/L × 0.01131 |
| Hb | g/L | g/dL | 0.1 | g/dL=g/L × 0.1 |

Abbreviations: BUN, blood urea nitrogen; SCr, serum creatinine; Hb, hemoglobin

Table S2. **Missingness of candidate continuous variables in the MIMIC-IV development cohort.**

| **Variables** | **Missing, n** | **Missing, %** |
| --- | --- | --- |
| Heart rate | 16 | 0.09 |
| SBP | 31 | 0.17 |
| DBP | 32 | 0.17 |
| SpO_2_ | 30 | 0.16 |
| RBC | 8 | 0.04 |
| SCr | 2 | 0.01 |
| BUN | 5 | 0.03 |
| Hb | 6 | 0.03 |
| PLT | 8 | 0.04 |
| WBC | 10 | 0.05 |
| PT | 1,162 | 6.27 |

Abbreviations: SBP, systolic blood pressure; DBP, diastolic blood pressure; SpO_2_, oxygen saturation; RBC, red blood cell count; SCr, serum creatinine; BUN, blood urea nitrogen; Hb, hemoglobin; PLT, platelet count; WBC, white blood cell count; PT, prothrombin time

Table S3. **Prespecified hyperparameter search space for candidate machine learning algorithms.**

| **Algorithm** | **Hyperparameter** | **Search space** |
| --- | --- | --- |
| Logistic regression | Inverse of regularization strength (C) | 0.01–10 |
|  | Tolerance (tol) | 10^-9^–0.1 |
|  | Maximum iterations (max_iter) | 10–500 |
|  | Solver (solver) | lbfgs, saga |
| XGBoost | Objective function (objective) | binary:logistic, reg:linear, reg:logistic |
|  | Learning rate (learning_rate) | 0.001–2 |
|  | Maximum tree depth (max_depth) | 1–20 |
|  | Minimum child weight (min_child_weight) | 1–20 |
|  | L2 regularization term (reg_lambda) | 0.001–5 |
| LightGBM | Boosting type (boosting_type) | gbdt, dart, goss |
|  | Learning rate (learning_rate) | 0.001–2 |
|  | Number of leaves (num_leaves) | 1–200 |
|  | Maximum tree depth (max_depth) | -1–20 |
|  | Number of trees (n_estimators) | 1–200 |
| Random forest | Number of trees (n_estimators) | 1–200 |
|  | Maximum tree depth (max_depth) | 1–20 |
|  | Split criterion (criterion) | gini, entropy |
|  | Minimum impurity decrease (min_impurity_decrease) | 0–20 |
| GNB | Variance smoothing (var_smoothing) | 10^-9^–0.1 |

**Abbreviations:** XGBoost, extreme gradient boosting; LightGBM, light gradient boosting machine; GNB, Gaussian Naive Bayes.

Table S4. **Comparison of the nine selected predictors between survivors and non-survivors in the external validation cohort.**

| **Variable** | **Overall (n = 314)** | **Survivors (n = 278)** | **Non-survivors  (n = 36)** | ***P* value** |
| --- | --- | --- | --- | --- |
| Age, years | 76.00 (67.00, 82.00) | 75.00 (63.00, 82.00) | 81.00 (71.00, 85.00) | 0.008 |
| Heart rate, bpm | 93.00 (81.00, 107.00) | 93.00 (82.00, 107.00) | 92.00 (77.00, 105.00) | 0.819 |
| SBP, mmHg | 138.00 (120.00, 157.00) | 138.00 (122.00, 158.00) | 137.00 (99.00, 148.00) | 0.166 |
| WBC, *10^9^/L | 8.51 (6.09, 11.60) | 8.10 (5.83, 10.50) | 14.23 (10.32, 16.82) | <0.001 |
| Hb, g/dL | 11.57 ± 2.78 | 11.76 ± 2.72 | 10.11 ± 2.83 | <0.001 |
| BUN, mg/dL | 27.17 (18.77, 43.42) | 25.77 (17.37, 39.21) | 59.66 (37.25, 78.99) | <0.001 |
| PT, s | 12.90 (11.90, 14.40) | 12.60 (11.80, 14.00) | 16.90 (14.30, 23.30) | <0.001 |
| Glucose, mg/dL | 138.57 (107.76, 198.22) | 136.77 (106.32, 190.47) | 196.06 (132.27, 229.21) | 0.021 |
| Potassium, mmol/L | 4.01 (3.62, 4.45) | 3.99 (3.59, 4.36) | 4.67 (3.71, 5.09) | 0.004 |

Abbreviations: SBP, systolic blood pressure; WBC, white blood cell count; Hb, hemoglobin; BUN, blood urea nitrogen; PT, prothrombin time

Table S5. **Performance of the finalized XGBoost model after hyperparameter refinement in the development cohort.**

| **Model** | **Dataset** | **AUC (****95% CI)** | **Acc** | **Sens** | **Spec** | **PPV** | **NPV** | **F1 score** |
| --- | --- | --- | --- | --- | --- | --- | --- | --- |
| XGBoost | CV–Train | 0.749 (0.739–0.760) | 0.692 | 0.672 | 0.696 | 0.299 | 0.917 | 0.413 |
|  | CV–Val | 0.712 (0.690–0.734) | 0.676 | 0.623 | 0.686 | 0.277 | 0.905 | 0.383 |

Abbreviations: CV, cross-validation; CV–Train, model performance in the training sets during nested cross-validation; CV–Val, model performance in the validation sets during nested cross-validation; AUC, area under the receiver operating characteristic curve; CI, confidence interval; Acc, accuracy; Sens, sensitivity; Spec, specificity; PPV, positive predictive value; NPV, negative predictive value

Table S6. **External validation performance metrics of the final XGBoost model.**

| **Model** | **AUC (95% CI)** | **Cutoff** | **Acc** | **Sens** | **Spec** | **PPV** | **NPV** | **F1 score** |
| --- | --- | --- | --- | --- | --- | --- | --- | --- |
| **XGBoost** | 0.877 (0.820–0.933) | 0.157 | 0.790 | 0.778 | 0.791 | 0.326 | 0.965 | 0.459 |

Abbreviations: AUC, area under the receiver operating characteristic curve; Acc, accuracy; Sens, sensitivity; Spec, specificity; PPV, positive predictive value; NPV, negative predictive value

Table S7. **Cross-cohort comparison of the nine final predictors and in-hospital mortality between the development cohort and the external validation cohort.**

| **Variables** | **Development cohort (n = 18,526)** | **External validation cohort**  **(n = 314)** | **SMD** | ***P* value** |
| --- | --- | --- | --- | --- |
| Age, years | 73.95 (64.18, 82.68) | 76.00 (67.00, 82.00) | 0.164 | 0.637 |
| Heart rate, bpm | 86.00 (75.00, 101.00) | 93.00 (81.00, 107.00) | 0.364 | <0.001 |
| SBP, mmHg | 119.00 (104.00, 137.00) | 138.00 (120.00, 158.00) | 0.721 | <0.001 |
| Hb, g/dL | 10.50 (8.90, 12.20) | 11.60 (9.60, 13.40) | 0.417 | <0.001 |
| WBC, *10^9^/L | 10.50 (7.50, 14.80) | 8.51 (6.09, 11.60) | 0.416 | <0.001 |
| BUN, mg/dL | 28.00 (18.00, 46.00) | 27.17 (18.77, 43.42) | 0.043 | 0.827 |
| Glucose, mg/dL | 130.00 (104.00, 174.00) | 138.57 (107.76, 198.22) | 0.143 | 0.010 |
| PT, s | 14.90 (12.90, 18.80) | 12.90 (11.90, 14.40) | 0.596 | <0.001 |
| Potassium, mmol/L | 4.20 (3.80, 4.70) | 4.01 (3.62, 4.45) | 0.296 | <0.001 |
| In-hospital mortality, n (%) | 2986 (16.12) | 36 (11.46) | 0.135 |  |

**Abbreviations:** SMD, standardized mean difference; SBP, systolic blood pressure; Hb, hemoglobin; WBC, white blood cell count; BUN, blood urea nitrogen; PT, prothrombin time.

Table S8. **Sensitivity and specificity under alternative threshold selection strategies for the finalized XGBoost model in the development cohort.**

| **Threshold selection approach** | **Sensitivity** | **Specificity** |
| --- | --- | --- |
| Threshold targeting sensitivity = 0.90 | 0.900 | 0.369 |
| Threshold targeting specificity = 0.90 | 0.365 | 0.900 |

Supplementary Figure S1. **Benchmark comparison with established ICU severity scores in the development cohort.** (A) ROC curves of SOFA and APS III for predicting in-hospital all-cause mortality; (B) DCA curve comparing the net benefit of the final XGBoost model, SOFA, APS III, and the treat-all and treat-none strategies across threshold probabilities.
Abbreviations: SOFA, Sequential Organ Failure Assessment; APS III, Acute Physiology Score III; ROC, receiver operating characteristic; AUC, area under the receiver operating characteristic curve; DCA, decision curve analysis.

Supplementary Figure S2. **Restricted cubic spline analyses of the nine continuous predictors retained in the final model in relation to in-hospital mortality in the development cohort.** All spline terms were fitted using 4 knots placed at the 5th, 35th, 65th, and 95th percentiles, corresponding to 3 degrees of freedom. **Abbreviations**: BUN, blood urea nitrogen; WBC, white blood cell count; PT, prothrombin time; SBP, systolic blood pressure; Hb, hemoglobin; CI, confidence interval.
